# Relationship between Size Summation Properties, Contrast Sensitivity and Response Latency in the Dorsomedial and Middle Temporal Areas of the Primate Extrastriate Cortex

**DOI:** 10.1371/journal.pone.0068276

**Published:** 2013-06-28

**Authors:** Leo L. Lui, James A. Bourne, Marcello G. P. Rosa

**Affiliations:** 1 Department of Physiology, Monash University, Clayton, Victoria, Australia; 2 Monash Vision Group, Monash University, Clayton, Victoria, Australia; 3 Australian Regenerative Medicine Institute, Monash University, Clayton, Victoria, Australia; University of Salamanca- Institute for Neuroscience of Castille and Leon and Medical School, Spain

## Abstract

Analysis of the physiological properties of single neurons in visual cortex has demonstrated that both the extent of their receptive fields and the latency of their responses depend on stimulus contrast. Here, we explore the question of whether there are also systematic relationships between these response properties across different cells in a neuronal population. Single unit recordings were obtained from the middle temporal (MT) and dorsomedial (DM) extrastriate areas of anaesthetized marmoset monkeys. For each cell, spatial integration properties (length and width summation, as well as the presence of end- and side-inhibition within 15° of the receptive field centre) were determined using gratings of optimal direction of motion and spatial and temporal frequencies, at 60% contrast. Following this, contrast sensitivity was assessed using gratings of near-optimal length and width. In both areas, we found a relationship between spatial integration and contrast sensitivity properties: cells that summated over smaller areas of the visual field, and cells that displayed response inhibition at larger stimulus sizes, tended to show higher contrast sensitivity. In a sample of MT neurons, we found that cells showing longer latency responses also tended to summate over larger expanses of visual space in comparison with neurons that had shorter latencies. In addition, longer-latency neurons also tended to show less obvious surround inhibition. Interestingly, all of these effects were stronger and more consistent with respect to the selectivity for stimulus width and strength of side-inhibition than for length selectivity and end-inhibition. The results are partially consistent with a hierarchical model whereby more extensive receptive fields require convergence of information from larger pools of “feedforward” afferent neurons to reach near-optimal responses. They also suggest that a common gain normalization mechanism within MT and DM is involved, the spatial extent of which is more evident along the cell’s preferred axis of motion.

## Introduction

The responses of single units in the primate visual system summate over larger areas of visual field during the presentation of low contrast stimuli, in comparison with high contrast stimuli [1], [2], [3], [4], [5], [6]. In addition, surround inhibition of neuronal responses becomes more prominent, and more frequent across the population of cells, as the stimulus contrast increases [7]. At the same time, we know that increasing the stimulus contrast also leads to a reduction in response latencies throughout the visual system [8], [9], [10], [11], [12]. To date, correlations between receptive field size, contrast sensitivity and latency have been demonstrated for single neuron responses. Here, we look at this issue from a different perspective, asking whether these response properties co-vary within neuronal populations. Cells in the same cortical area differ with respect to the way they respond to changes to stimulus size and contrast [13], [14], [15], [16], [17], [18], and their response latencies encompass relatively broad ranges. This raises the question of whether there are systematic relationships between the spatial summation properties of different cells, their contrast sensitivities, and response latencies. For example, do cells with low and high contrast sensitivity differ in terms of their spatial summation properties? Is a neuron’s response latency related to its contrast sensitivity? These questions have implications for understanding the spatial and temporal dynamics of how large populations of neurons respond in concert, during viewing of natural scenes.

Interdependency between size summation properties and contrast sensitivity has been demonstrated for cells in the middle temporal extrastriate area (MT) [5]. In addition to their well-characterized selectivity for direction of motion [19], [20], MT cells often prefer stimuli of specific lengths and widths [17], [21], a characteristic that makes them suitable targets for the present investigation. For comparison, we studied cells in the dorsomedial area (DM), an extrastriate area that is similar to MT in terms of a dominant afferent input from layer 4 b of V1 [22], heavy myelination, and neuronal receptive field sizes [23], [24]. Despite these similarities, DM and MT are physiologically and anatomically distinct [25], [26], [27]. For example, unlike those in MT, DM neurons vary widely with respect to direction selectivity, but tend to be narrowly tuned for orientation [28]. Our results indicate that contrast sensitivity is related, in both areas, to size summation, alluding to common neural mechanisms. In addition, results in MT indicate that the shortest-latency responses to stimuli flashed within the receptive field are associated with the neurons that show the highest contrast sensitivity.

## Materials and Methods

### Ethics Statement

The experiments were approved by the Monash University Animal Experimentation Ethics Committee (Project Approvals: PHYS/2000/09, PHYS/2003/05, SOBSA/2006/10), which also monitored the welfare of the animals. All procedures followed the guidelines of the *Australian Code of Practice for the Care and Use of Animals for Scientific Purposes*. Data were collected from 18 adult New World monkeys (*Callithrix jacchus*, the common marmoset), as part of a series of experiments that also included single-unit recordings from other areas, and analyses of neuronal responses to other types of stimulus [17], [28], [29], [30], [31], [32], [33]. These animals were bred for the purpose of scientific research at the Australian National Primate Facility, sponsored by the National Health and Medical Research Council. They were housed with compatible animals in cages with 0.9 m^2^ floor space and 2 m in height, with daily access to outside runs (1.4 m^2^ floor space and 2 m in height). Throughout their life they had *ad libitum* daily access to water and balanced nutrient pellets, as well as fruit, vegetables and meal worms on different days of the week. In addition to the outside runs and diet rotation, environmental enrichment was provided in the family cages, in the form of ropes, ladders, hanging toys, bamboo stems, and other substrates to encourage diverse motor activities. Two to four weeks before the electrophysiological experiments they were transported to Monash University, where they were housed in 2 m^3^ cages (1 m^2^ floor area, and 2 m high). Animals were housed in pairs wherever a compatible partner was available, and were always within visual and acoustic range of other individuals of the same species. The same types of indoor enrichment and diet described above were available at this location, and their health was monitored on a daily basis. The animals were anaesthetized for the entire period of the electrophysiological experiments, and were killed by barbiturate overdose at the end of the recording sessions without recovering consciousness.

### Preparation

The surgical preparation and the procedures for recording and visual stimulation have been described in detail [34]. Anesthesia was induced with ketamine (50 mg.kg^−1^) and xylazine (3 mg.kg^−1^), allowing a tracheotomy, cannulation of the saphenous vein, and a craniotomy. The dura mater overlying the dorsal cortical surface was removed, and the cortex covered with a thin layer of silicone oil in order to prevent desiccation. After all surgical procedures were completed, the animal was administered an intravenous infusion of pancuronium bromide (0.1 mg.kg^−1^.h^−1^), combined with sufentanil (6 µg.kg^−1^.h^−1^) and dexamethasone (0.4 mg.kg^−1^.h^−1^), in a saline/glucose solution, which induced muscular paralysis while maintaining anesthesia. The animal was artificially ventilated with a gaseous mixture of nitrous oxide and oxygen (7∶3). The level of anesthesia was monitored using electrocardiogram, blood pressure, SpO_2_, and the level of cortical spontaneous activity. Administration of atropine (1%) and phenylephrine hydrochloride (10%) eye drops resulted in mydriasis and cycloplegia. Appropriate focus and protection of the corneas from desiccation were achieved by means of hard contact lenses, which brought into focus the surface of a computer monitor located 40 cm in front of the animal. Visual stimuli were presented to the eye contralateral to the hemisphere from which the neuronal recordings were obtained.

### Recording Sites

Parylene-coated tungsten microelectrodes with exposed tips of 10 µm were directed towards areas MT and DM based on stereotaxic coordinates and sulcal morphology. Provisional attribution of recording sites to MT during the experiment was based on mapping of multiunit receptive fields, using electrodes that penetrated vertically: a dorsoventral movement of the electrodes in the brain is expected to result in a gradual shift in the position of the MT receptive fields, from the lower quadrant towards the upper quadrant, and a gradual decrease in the eccentricity of receptive fields [35]. This trend, together with the obvious direction selectivity of neurons, allowed us to estimate the dorsal and ventral borders of MT during the recording session. The initial attribution of recording sites to DM was also initially determined by mapping of multiunit receptive fields, conducted at the beginning of the experimental sessions. In this case, sequences of recording sites starting in dorsal V2 (second visual area) and moving progressively more anterior, across DM, revealed receptive fields that drift gradually from the horizontal meridian of the visual field to the vicinity of the vertical meridian [36]. Confirming previous reports [37], [38], we found that, depending on the mediolateral level, a rostral progression of recording sites within DM resulted in receptive fields that either reverted towards the lower visual field, or moved into the upper field. The present sample of DM recordings was concentrated on the part of DM located on the dorsal surface near the midline, resulting in receptive fields in the lower visual field. In both DM and MT, we obtained samples of receptive fields centred at a similar range of eccentricities (5°–20°). Confirmation of the location of the recording sites in both areas was based on histological examination of the electrode tracks, relative to myeloarchitectural criteria [23], [24].

### Electrophysiological Recordings

Amplification and filtering of the electrophysiological signal was achieved via a Model 1800 Microelectrode AC amplifier (AM Systems) and a 50 Hz eliminator (HumBug, Quest Scientific). The processed signal was fed into a waveform discriminator (SPS-8701, Signal Processing Systems), allowing the isolation of single unit signals by means of a template-matching algorithm. The neural activity was continuously monitored by means of loud speakers, an oscilloscope (raw signal) and computer displays (processed signal, corresponding to the isolated units under investigation). For quantitative analyses, the spike trains were collected via a high-fidelity interface (ITC-16, Instrutech) into a Macintosh Power PC computer, which controlled the visual stimulus generation and displayed the accumulated peristimulus time histograms (PSTHs).

The initial exploration of the receptive field boundaries was conducted using hand-held stimuli moved at various speeds, orientations and directions of motion across the screen of a 20-inch monitor (resolution 1,024×768 pixels). Estimates of receptive field borders were then refined by presenting computer-generated bars, gratings and flashing spots, while listening to the cell’s activity. Following the determination of the receptive field centers (points of maximal response), neuronal response properties were studied quantitatively using computer-controlled stimuli. The experiments consisted of two steps, carried out sequentially for each DM or MT neuron. First, we obtained estimates of the cell’s size selectivity and response latency characteristics, using high contrast (60%) gratings of optimal spatial and temporal frequency and optimal direction of motion. Then, the contrast sensitivity functions for the same cells were measured in tests where the contrast was the only variable manipulated, while spatiotemporal characteristics, direction and size were kept constant at near-optimal values (as determined in the course of the initial tests).

Each condition was presented a minimum of eight times, in randomized order within each block. An inter-trial interval of at least 4 s, during which the grey screen was presented, separated trials. The stimuli for all quantitative tests consisted of rectangular patches of drifting gratings presented against a uniform grey background. The grey background had luminance equivalent to the average across the gratings (which was in the low photopic range, 2.7 cd.m^−2^). Each trial started with a 0.5 s presentation of the grey screen, during which measurements of spontaneous activity were obtained. Drifting gratings were then presented for 2 s at constant speed, with the phase at the centre point of the stimulus randomized between trials, drawn from a distribution including ¼ cycle steps. In the majority of the tests, encompassing cells in both DM and MT, the presentation of the gratings was “ramped”, with the contrast increasing from zero to the desired value over the first 500 ms of presentation. This approach was chosen in order to minimize transient responses to the sudden appearance of the grating on the screen (thus dissociating sensitivity to flashes from contrast sensitivity per se). However, this had the obvious consequences of increasing the estimates of response latency because of the additional time required for the stimulus to reach threshold contrast, and possibly allowing cells with higher contrast sensitivity to respond earlier. Thus, in order to investigate the relationships between size summation, contrast sensitivity and latency, in a subset of MT cells the gratings were “flashed” immediately from the first frame of presentation (hence isolating the component due to conduction along the visual pathway). Only cells using “flashed” presentation were used to assess relationships with latency.

The tests conducted for each cell followed the same sequence. First, tests were performed to determine optimal values of direction of motion, spatial frequency (range tested 0.08–2.4 cycles.deg^−1^) and temporal frequency (0.18–10.9 Hz) for each neuron, using 60% contrast gratings. This range was chosen empirically, to encompass optimal values for most, if not all cells in the sampled regions of DM and MT [28], [32]. Once optimal spatiotemporal parameters were determined, we performed size selectivity tests, in which the length and width of the gratings were manipulated independently [17]. For each cell, five values of length and width were tested, resulting in 25 stimulus conditions. These included values that were smaller than the minimum response receptive fields of most MT and DM neurons (2°, 4°), values that approximately coincided with the typical sizes of these receptive fields (8°, 16°), and values that were larger than the vast majority of receptive fields (30°). The different grating dimensions were achieved by electronic “masking” of a single large stimulus. Throughout this paper references to the length of the grating stimulus indicate the dimension along which the grating elements had constant luminance, while along the width dimension the grating luminance changed according to a sine-wave function. The direction of motion was always parallel to the width of the grating. Finally, gratings of 5 different luminance contrasts were presented, ranging from 2% to 95% at the peak (constant-contrast) part of the presentation. In these tests the gratings had near optimal direction, spatial frequency, temporal frequency, length and width, as determined by the initial tests (i.e., at 60% contrast).

### Histology

At the end of the experiment the animal was administered an overdose of sodium pentobarbitone and perfused transcardially with 0.9% saline, followed by 4% paraformaldehyde in 0.1 M phosphate buffer (pH 7.4). After cryoprotection by increasing concentrations of sucrose and sectioning at 40 µm, alternate slides were stained for Nissl substance, using cresyl violet, and for myelin, using the Gallyas [39] method. Electrode tracks were reconstructed with the aid of small electrolytic lesions (4 µA, 10 s), which were placed at various sites during the experiment. Only cells confirmed as belonging to MT and DM, on the basis of the patterns of myelination [23], [24], were included in the present report.

### Data Analysis

The responses of each cell were converted into PSTHs with a 10 ms bin width, which formed the basis of all subsequent analysis. A single trial response was computed as the mean firing rate over the entire duration of stimulus (2 sec). Spontaneous activity was calculated from the mean firing rate during the 500 ms before stimulus onset. We only included cells which responded at a level at least two standard deviations above the mean spontaneous activity.

Both size summation properties and contrast response functions were determined by fitting parametric models (see below) using the Matlab function “lsqcurvefit” (MathWorks, Natick, MA). Using this approach, the best fit for each neuron was obtained by minimizing the sum-squared error between the neuronal response and the values obtained by the function. Curve fitting was based on the entire matrix of single trial responses, rather than the mean responses to each stimulus condition. The fittings were always constrained by the requirement that the resulting curves should cross the level of spontaneous activity at zero values of length, width or contrast [29]. Both parametric and non-parametric statistical tests were used in the analyses, as specified in “Results”.

Since the length and width summation properties of neurons can be interdependent [17], for each cell we fitted a 2 dimensional Gaussian to the matrix of responses as a function of grating length and width, after subtraction of the spontaneous activity:
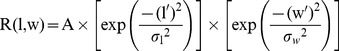
(1)


Where
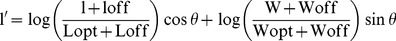
(2)


Where
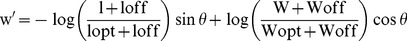
(3)


**Figure 1:**
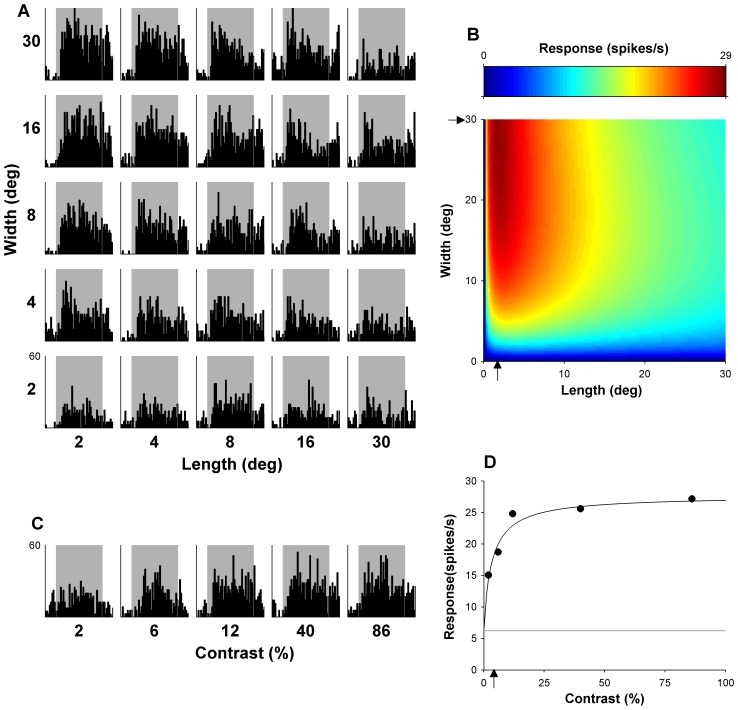
Example MT neuron. (A) Shows the matrix of PSTHs representing trials to all lengths and widths presented. The vertical scale, displaying response rate in spike/sec is located on the bottom left plot; the same scale applies to all histograms. (B) Shows the optimal fit of equation (1), which estimates response with respect to length and width. The arrows indicate estimates of optimal length and width, given by fitted parameters. This neuron is end-inhibited (EI) but shows no evidence of side inhibition when probed with stimuli covering up to 30° of visual angle (NSI_15_). (C) Displays peri-stimulus time histograms representing response over time to gratings of varying contrasts, shown with the same conversions as in (A). (D) Show the mean responses with respect to varying contrasts over the entire 2 sec presentation, fitted with equation (4). The arrow indicates C_50_. Thin grey line indicates mean spontaneous activity measured in the 500 ms before the onset of stimuli.

**Figure 2:**
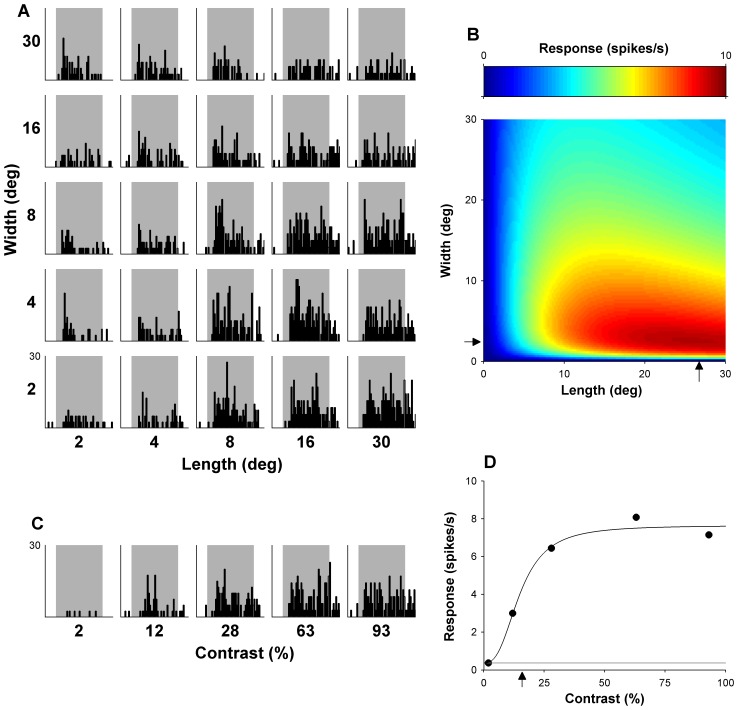
Responses of an example DM neuron, illustrated with the same conventions used in Figure 2. (A) Shows the matrix of PSTHs obtained by presentation of all lengths and widths tested, and (B) the shows the optimal fit of equation (1), with estimates of optimal length and width (arrows). This neuron is side-inhibited (SI) but shows no evidence of end inhibition when probed with stimuli covering up to 30° of visual angle (NEI_15_). (C) Displays PSTHs representing response over time to gratings of varying contrasts. (D) Show the mean responses with respect to varying contrasts, and an estimate of C_50_ (arrow).

The function is a variant of the model used in [17]. Here, R(l, w) represent the response with respect to length (l) and width (w), while A, l_opt_, w_opt_, σ_l_, σ_w_, l_off_, w_off_, and θ are free parameters. Parameter A accounts for the maximum response of the cells above spontaneous activity, and optimal length and width are given by l_opt_ and w_opt_, respectively, which were constrained to a maximum value of 30° (the maximum length and width of the tested grating patches). Parameters σ_l_ and σ_w_ determined the width of the curve for each dimension. The offset parameters, l_off_ and w_off_, are necessary for two reasons. First, they keep the logarithm from becoming undefined as the stimulus size approaches zero. Second, they also allow the rate of increase and decrease to deviate from a strict log-Gaussian function, hence affecting the shape of the tuning curve. Parameter θ represents rotation of the model around its peak, allowing for interaction between length and width. This function provided good fits to the data, with median R^2^ values of 0.93 and 0.92 for our sample of MT and DM cells respectively. Most importantly, it provided reliable parametric estimates of the optimal grating sizes for all of our neurons (see [17] for discussion). Responses and optimal fits to responses are illustrated in Figures 1 and 2.

In order to determine whether inhibition occurred at longer lengths and widths, confidence intervals for parameter estimates were computed from the Jacobian matrix and the residuals using the Matlab function “nlparci”. If the 95% confidence interval prediction for the parameter l_opt_ (optimal length) did not overlap with 30° (maximum patch length tested), the cell was classified as end-inhibited (EI). Conversely, if the 95% confidence interval prediction for parameter w_opt_ (optimal width) did not extend to 30°, the cell was classified as side-inhibited (SI). Cells that were not EI or SI were classified as non-end inhibited by stimuli extending 15° on either side of the receptive field centre (NEI_15_) or non-side inhibited by stimuli extending 15° on ether side of the receptive field centre (NSI_15_). This nomenclature reflects the need for caution in the interpretation of results, as the entire visual field could not be stimulated using a CRT-based system; thus, it is possible that NEI_15_ and NSI_15_ cells would have revealed some degree of surround inhibition, had the stimuli been extended even further. However, we regard this caveat as unlikely to have affected our main conclusions regarding the relationship between strength of end- and side-inhibition and other variables. Here, it is important to recall that the maximum stimulus size used in the present experiment is at least 50% larger than the known excitatory receptive field sizes of cells in DM and MT at the eccentricities sampled in our experiments (<15° [23], [24]). It has been established that many cells in both DM and MT show facilitatory surrounds outside the borders of the classical receptive fields [28], [30], [40], therefore, size preferences of <15° should be interpreted as facilitation from beyond the classical receptive field [28] rather than the size of the receptive field itself. Finally, despite these caveats, we note that the majority of neurons classified as NEI_15_ and NSI_15_ reached response plateaus at stimulus size values well below the 30° limit of our presentation system (e.g. Fig. 1). Extending the stimuli beyond 30°, although possible through the use of a spherical projection system [41], would have resulted in less precise control of stimulus contrast, a key requirement for the present analyses.

To determine contrast sensitivity, the responses of cells to different contrasts were fitted (following subtraction of spontaneous activity) with the following function:
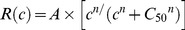
(4)where R(c) represents responses with respect to contrast (c). There were three free parameters: A, representing the maximal response, n, the exponent indicating the slope, and C_50_, the half saturation contrast (See Figs. 1 and 2). This function provides a good fit to contrast response functions of cells from visual cortex of both cat and monkey [42], [43], [44] Our data were no exception: the MT and DM data had median R^2^ values of 0.97 when fitted with this function.

**Figure 3:**
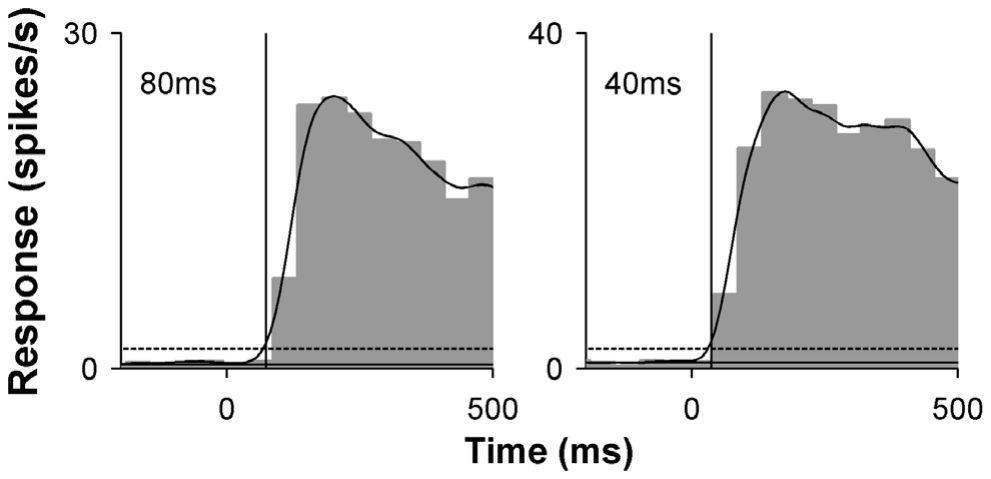
Two examples of latency calculation for MT neurons. For each example, grey bars demonstrate the “grand PSTHs” (representing the neuron’s response to gratings of optimal orientation, spatial and temporal frequency, and various sizes), with respect to stimulus onset (0 ms). The curved line illustrates the spike density function for which the latency was calculated. The solid horizontal line denotes the average spontaneous activity while the dotted line is one standard deviation above the spontaneous activity. The time at which these spike density function crosses this threshold represents an estimate of the latency of a particular cell (indicated by the vertical line and number). Both examples were presented via the “flashed” method.

A standardized estimate of neuronal response latency of MT cells to “flashed” gratings was determined using combined data obtained in all trials in the tests aimed at determining size selectivity. As explained above, these trials (25 conditions x 8 repeats, = 200 trials) corresponded to presentations of 60% contrast gratings at optimal direction, spatial and temporal frequency. The neural activity obtained in these tests was combined into a “grand PSTH” [33], which was used to determine response latency. As shown in Figure 3 the spike trains were convolved with a Gaussian kernel (σ = 20 ms), in 10 ms steps, and the latency was estimated as the time at which the resulting spike density function first exceeded one standard deviation above spontaneous activity.

Both parametric and non-parametric statistical tests, depending on the distribution of the data, were used to compare means and evaluate correlations between two variables. To test whether two variables had any effect on a third variable, we fitted linear models to our data. We added one free parameter at a time to the model which represented each variable and possible interaction effects. Sequential F-tests were used to evaluate whether the each extra free parameter in the model provided a better fit to the data. A significant result (P<0.05) would imply that a particular variable was significantly related to the dependent variable.

## Results

### Comparison of Size Summation Properties

We characterized the size summation properties of 157 MT neurons and 99 DM neurons using high contrast (60% gratings). Previous studies have reported on size summation characteristics of cells in these areas [17], [28]. Here, in order to allow for a more direct comparison, we applied the same methods of analysis to samples obtained under identical experimental conditions, and only included receptive fields covering the same range of eccentricities. Therefore, we only included MT data that were obtained with “ramped” stimulus presentation (N = 79), as all DM samples were gathered using this method. This analysis supported the view that cells in these areas have distinct size selectivity properties.

**Figure 4:**
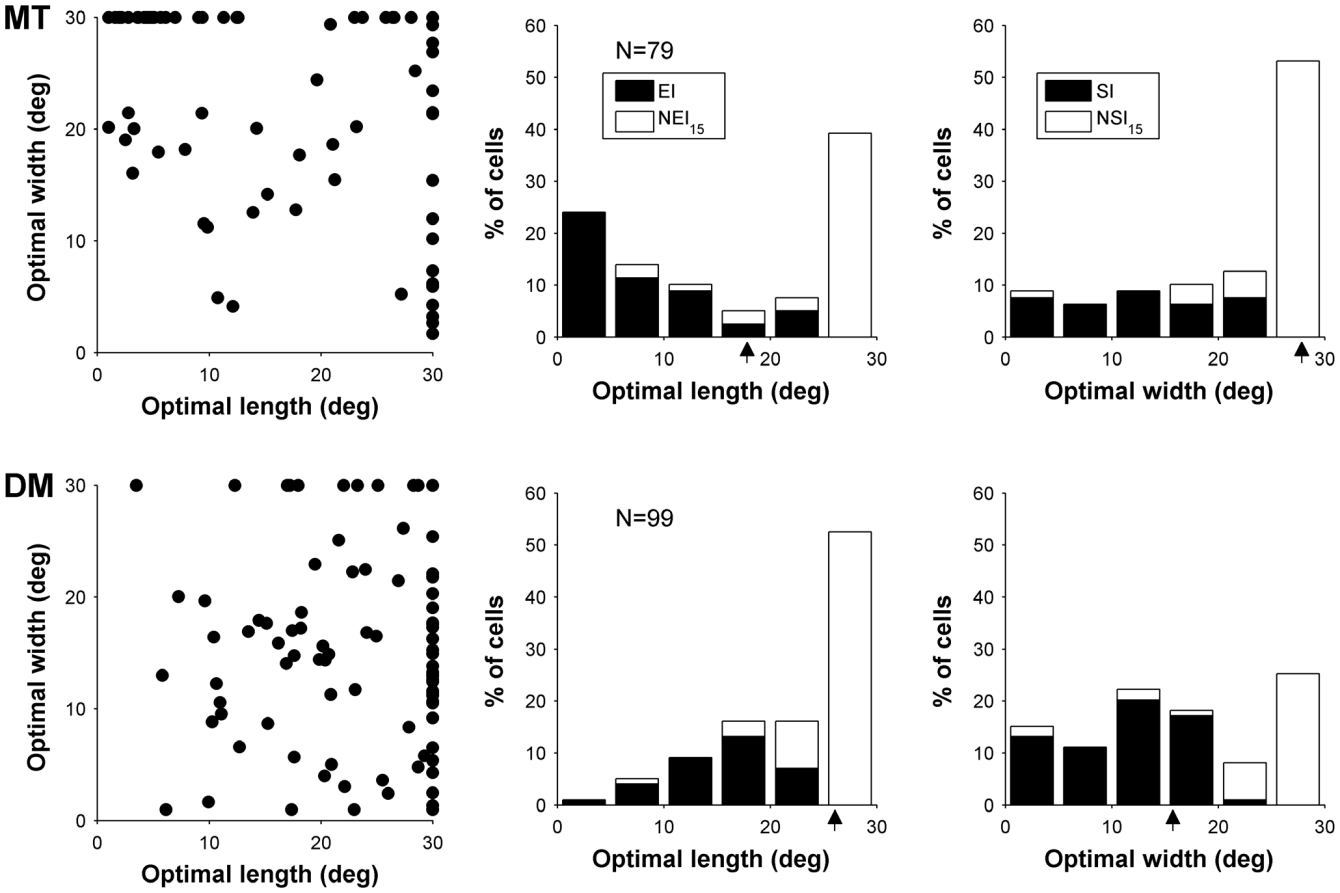
Length and width summation properties of cells in MT (top row) and DM (bottom row). Left column: the preferred length and width for each cell. Comparison of the distribution of optimal lengths (middle column) and optimal widths (right column) are also shown. For all histogram arrows denote the median of each distribution. DM cells prefer significantly longer gratings while MT cells prefer wider gratings (p<0.05).

Examples of the dependence of responses of typical MT and DM neurons on grating length and width are illustrated in Figures 1 and 2, respectively. Optimal fits of equation (1) to these data are presented in part B of these figures. As explained in the Materials and Methods, the optimized values of l_opt_ and w_opt_ yielded estimates of optimal length and width, and the confidence intervals of these free parameters were used to determine the presence of end- and side-inhibition within the stimulated zone surrounding the receptive field centre (to a maximum of 30°×30°). Figure 4 (left) summarises the preferred length and width of gratings for our sample of MT (top) and DM (bottom) neurons. On average, MT neurons preferred significantly shorter grating patches, in comparison with DM cells (Optimal length; median [MT] = 17.8°; median [DM] = 26.0°; Wilcoxon rank sum test, P = 0.0001; Fig. 4 middle column). Correspondingly, a significantly larger proportion of MT cells showed end-inhibition when stimulated with gratings up to 30° in size (52%), in comparison with those in DM (34%; χ^2^(1) = 5.6, P = 0.018). The opposite effect was observed when preference for the width of the stimulus was tested: MT neurons preferred wider patches (i.e., consisting of more cycles of the grating moving in file) in comparison with DM neurons (Optimal width; median [MT] = 27.7°; median [DM] = 15.6°; Wilcoxon rank sum test p = 0.0004; Fig. 4 right column). The proportion of NSI_15_ cells was also lower in MT (37%) than in DM (63%; χ^2^(1) = 4.5, P = 0.001). In summary, MT neurons tend to respond maximally to gratings that are relatively short and wide, while those in DM prefer gratings that are long and narrow.

### Relationship between Size Selectivity and Contrast Sensitivity

**Figure 5:**
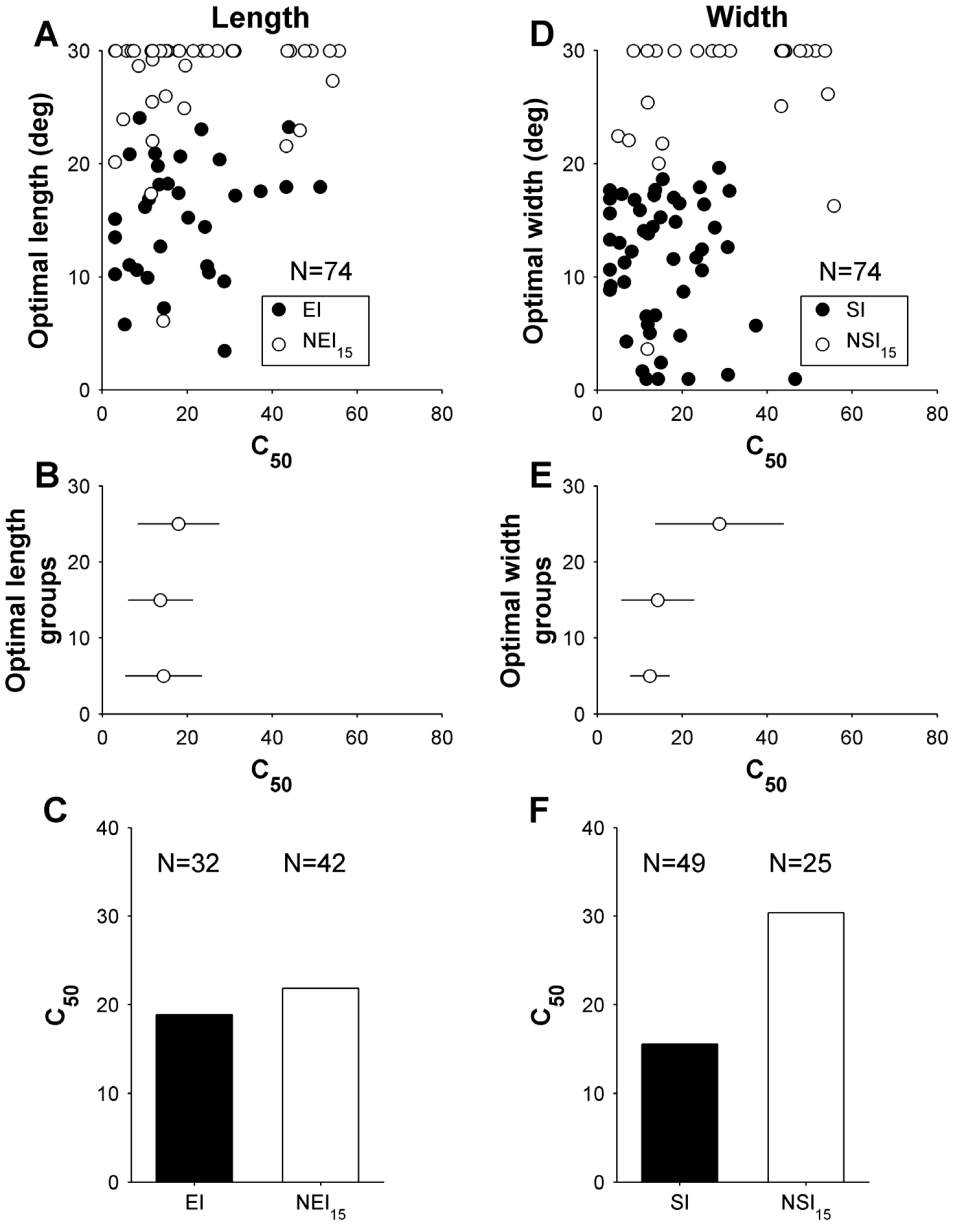
Relationship between contrast sensitivity (half-saturation contrast, C_50_) and the spatial properties of the receptive fields in area DM. Separate analyses are presented for the length (left column) and width (right column) dimensions of the receptive field. Top row shows the relationship between the optimal length (A) and width (D) of the stimulus, and contrast sensitivity (C_50_). Data from cells that showed significant spatial inhibition upon presentation of stimuli up to 30° in length or width are indicated by filled circles (end inhibition in panel A, side inhibition in panel D). The middle row summarizes the data shown in the top row, by grouping neurons according to the preferred length (B) and width (E) in three groups, according to optimal size (<10°, 10–20° and >20°). The data points are medians for these groups, and error bars represent the inter quartile ranges. Bottom row illustrates the mean C_50_ for DM cells. Black bars represent means for EI and SI cells (in C and F, respectively), and white bars represent the means for NEI_15_ and NSI_15_ cells.

**Figure 6:**
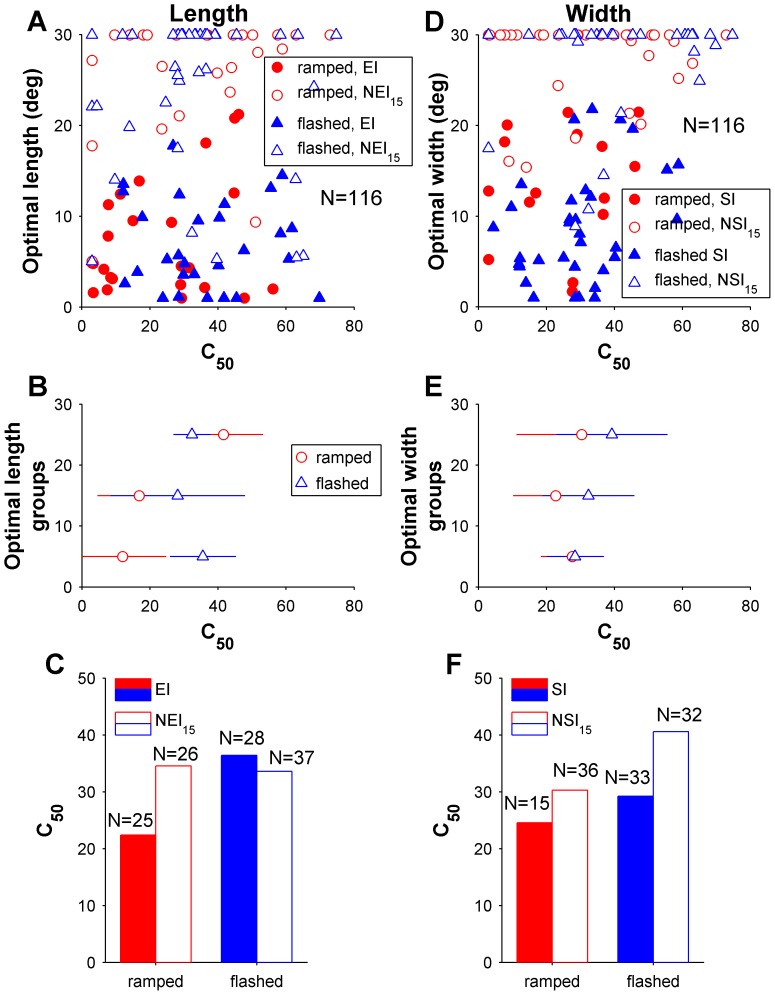
Relationship between contrast sensitivity (half-saturation contrast, C_50_) and the spatial properties of the receptive fields in area MT. Top row shows the relationship between optimal length (A), width (D) and C_50_, with filled symbols indicating cells that displayed significant end and side-inhibition (in A and D, respectively) upon presentation of stimuli up to 30° in size. Results from cells tested with “flashed” gratings are indicated by blue triangles, and those from cells tested with “ramped” gratings by red circles. The middle row summarizes the data shown in the top row, by grouping neurons according to the preferred length (B) and width (E), in three groups according to optimal size (<10°, 10–20° and >20°). The data points are medians for these groups, and error bars represent inter quartile ranges. Bottom row illustrates the mean C_50_ for cells in MT, grouped according to their method of presentation and spatial inhibition properties along the length (C) and width (F) dimensions of the receptive field.

In this section we consider whether the size summation properties of different cells are related to their contrast sensitivity. Figures 5 and 6 illustrate aspects of the relationship between size summation properties and contrast sensitivity. A total of 116 MT and 79 DM neurons were included, for which both the size selectivity test and the contrast sensitivity test were completed. Measures of preferred stimulus size were described above, and C_50_ was used as a measure of contrast sensitivity (Figs. 1 and 2).

In DM the relationship between preferred length and contrast sensitivity was not significant (Spearman’s r = 0.17, P = 0.16; Fig. 5A, B), and no difference in median C_50_ was found when cells were classified as EI or NEI_15_ (Wilcoxon rank sum P = 0.58; Fig. 5C). In contrast, a significant positive correlation was found between preferred width and C_50_ (Spearman’s r = 0.33, P = 0.003), as illustrated in Figure 5 (D, E). This relationship was supported by that fact that the SI cells had significantly lower C_50_, in comparison with NSI_15_ cells (Wilcoxon rank-sum test, P = 0.0007; Fig. 5F).

Results obtained in area MT resembled those in DM in that there was no significant main effect of preferred length on contrast sensitivity (F = 2.25, P = 0.14; Fig. 6A, B). There was, in addition, no main effect of method of presentation (“ramped” versus “flashed”) on contrast sensitivity (F = 3.2, P = 0.075). However, the results indicated a significant interaction effect between preferred length and method of presentation, in relation to contrast sensitivity (F = 7.4, P = 0.008). This is evidenced in Figure 6B by a significant positive correlation between optimal length and C_50_, which was present when MT cells were tested with “ramped” stimuli (Spearman’s r = 0.35, P = 0.01), but not with “flashed” stimuli (Spearman’s r = −0.06, P = 0.61). Using evidence of end-inhibition in the neighbourhood of the receptive field as a measure of length selectivity reveals a parallel result (Fig. 6C): there was no effect of end-inhibition on contrast sensitivity (F = 1.45, P = 0.23) and no significant main effect for method of presentation (F = 2.9, P = 0.09), but there was a significant interaction effect (F = 8.45, P = 0.004). EI cells were more sensitive to lower contrasts in the “ramped” condition (Wilcoxon rank sum P = 0.04; Fig. 6C), but not the “flashed” condition (P = 0.51).

The relationship between width summation and contrast sensitivity in MT was simpler, and resembled the results obtained in DM. Cells that preferred narrow gratings tended to be more sensitive to low contrast (F = 6.06, P = 0.015; Fig. 6D, E). A main effect for method of presentation on C_50_ was also found (F = 6.86, P = 0.01), but no interaction was present (F = 0.83, P = 0.36), indicating the relationship between preferred width and C_50_ was applicable to both “flashed” and “ramped” gratings. Analysis of the data according to the presence or absence of significant side-inhibition in the neighbourhood of the receptive field supported these findings (Fig. 6F), with significant main effects being found for presence of side-inhibition (F = 4.3, P = 0.04) and method of presentation (F = 5.3, P = 0.02), but no interaction being evident between these factors (F = 0.60, P = 0.44).

### Relationship between Contrast Sensitivity and Response Latency in MT

**Figure 7:**
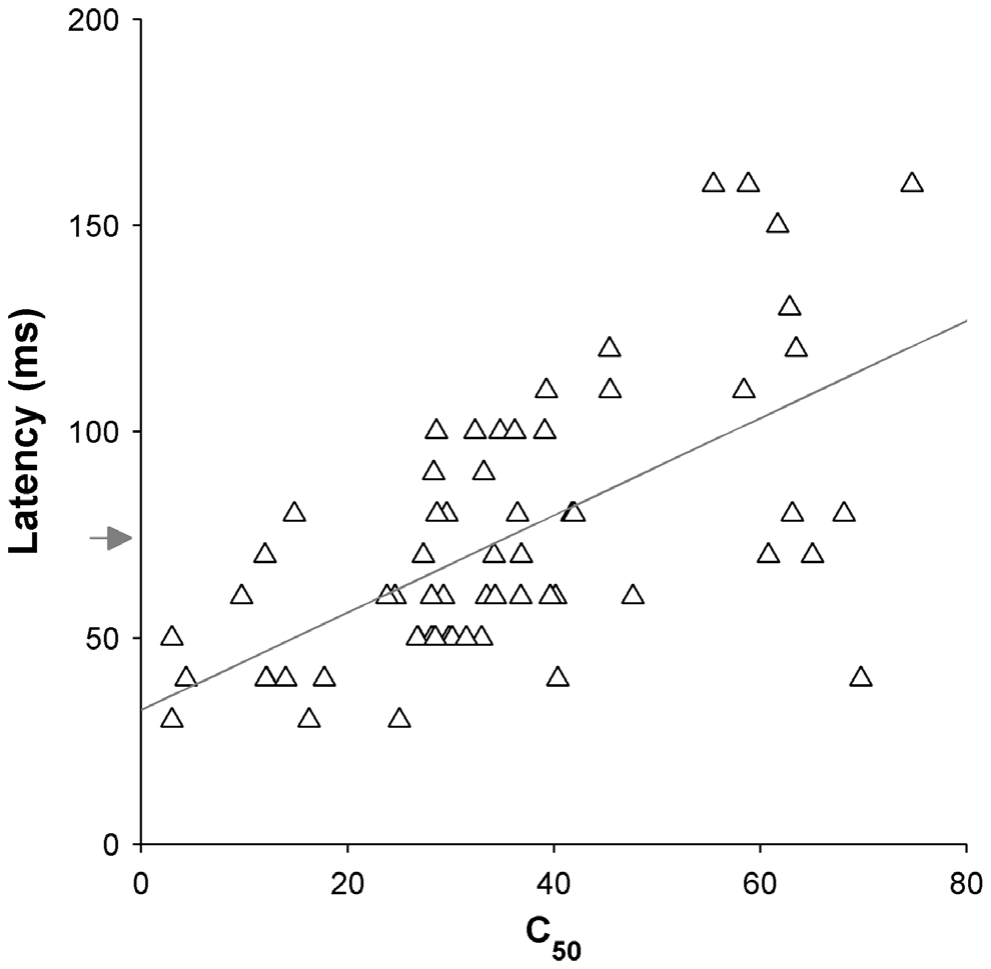
Relationship between contrast sensitivity and latency in MT. Only data using the “flashed” method of presentation is included here. Arrow indicates mean latency and line indicates the best linear fit.

We asked whether there is a cross-population relationship between contrast sensitivity and response latency, with the hypothesis that neurons that are highly sensitive to contrast also tend to respond earlier (i.e., to have shorter neural latencies) upon the presentation of a same stimulus. For each cell a standardised value of response latency was obtained upon presentation of 60% contrast gratings of optimal spatial and temporal frequency (Fig. 3), and C_50_ was used as a measure of contrast sensitivity (Figs. 1D, 2D). To eliminate the possible confounds, analysis here was restricted to neurons that were presented with “flashed” gratings; therefore, the analysis presented in Figure 7 includes data from 74 MT neurons. A significant correlation was found (Spearman’s r = 0.63 P<0.00001), where cells which responded earlier were also more sensitive to low contrasts. The mean latency in response to “flashed” gratings in MT was 74.2±4.1 ms, which compared well with values obtained in the macaque [45], [46], although many of the cells with high contrast sensitivity responded with latencies under 50 ms.

### Relationship between Preferred Stimulus Size and Response Latency in MT

**Figure 8:**
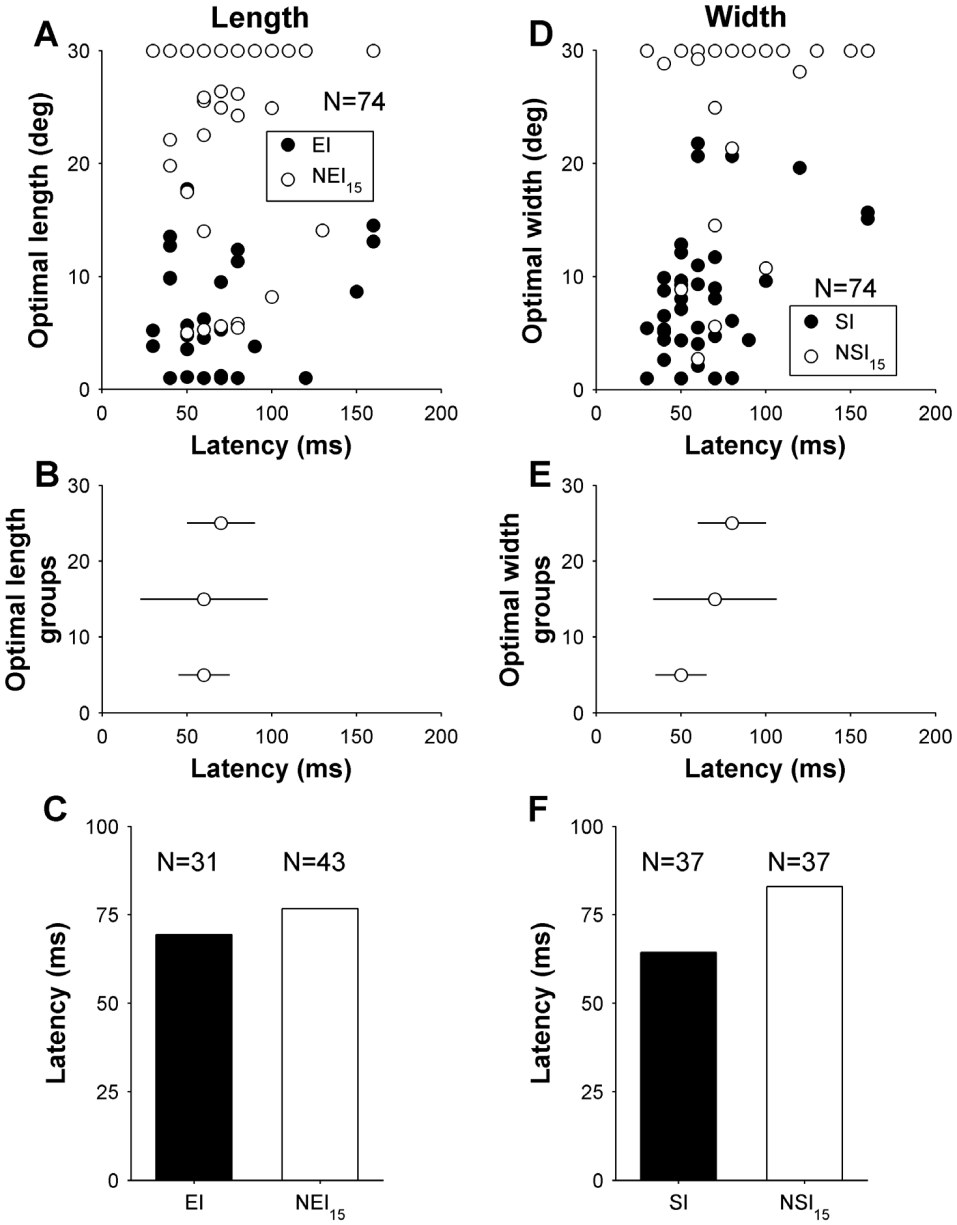
Relationship between latency, length (left column) and width (right column) selectivity in area MT. Top row shows the relationship between optimal length (A), width (D) and latency, with filled symbols indicating cells that displayed significant end and side-inhibition (in A and D, respectively) upon presentation of stimuli up to 30° in size. The middle row summarizes the data shown in the top row, by grouping neurons according to the preferred length (B) and width (E), in three groups according to optimal size (<10°, 10–20° and >20°). The data points are medians for these groups, and error bars represent inter quartile ranges. Bottom row illustrates the mean latency for cells in MT, grouped according to spatial inhibition properties along the length (C) and width (F) dimensions of the receptive field. Only data using the “flashed” method of presentation is included here.

In this section we consider whether the size summation properties of different cells were related to their response latencies. Our hypothesis was cells that summate across larger expanses of visual space take longer to respond, as they are likely to represent higher-order levels of hierarchical processing within the same area [47]. Again, this analysis was restricted to the 74 MT cells with “flashed” gratings. We analysed whether both the optimal length and width of visual stimuli, and the presence of end- and side-inhibition in the region of the visual field immediately surrounding the receptive field were related the response latencies of different cells. No significant relationship was found between preferred length and latency (Spearman’s r = 0.23, P = 0.053; Fig. 8A and B), and, correspondingly, the latencies of EI cells were not significantly different to those of NEI_15_ cells (Wilcoxon rank-sum test: P = 0.09; Fig. 8C). However, there was a significant relationship between optimal width and latency: cells that preferred narrow gratings responding significantly earlier than cells that preferred wide gratings (Spearman’s r = 0.51, P<0.00001; Fig. 8D and E). In addition, SI cells also tended to respond earlier than NSI_15_ cells (Wilcoxon rank-sum test: P = 0.0008; Fig. 8F).

### Effect of Firing Rates

**Figure 9:**
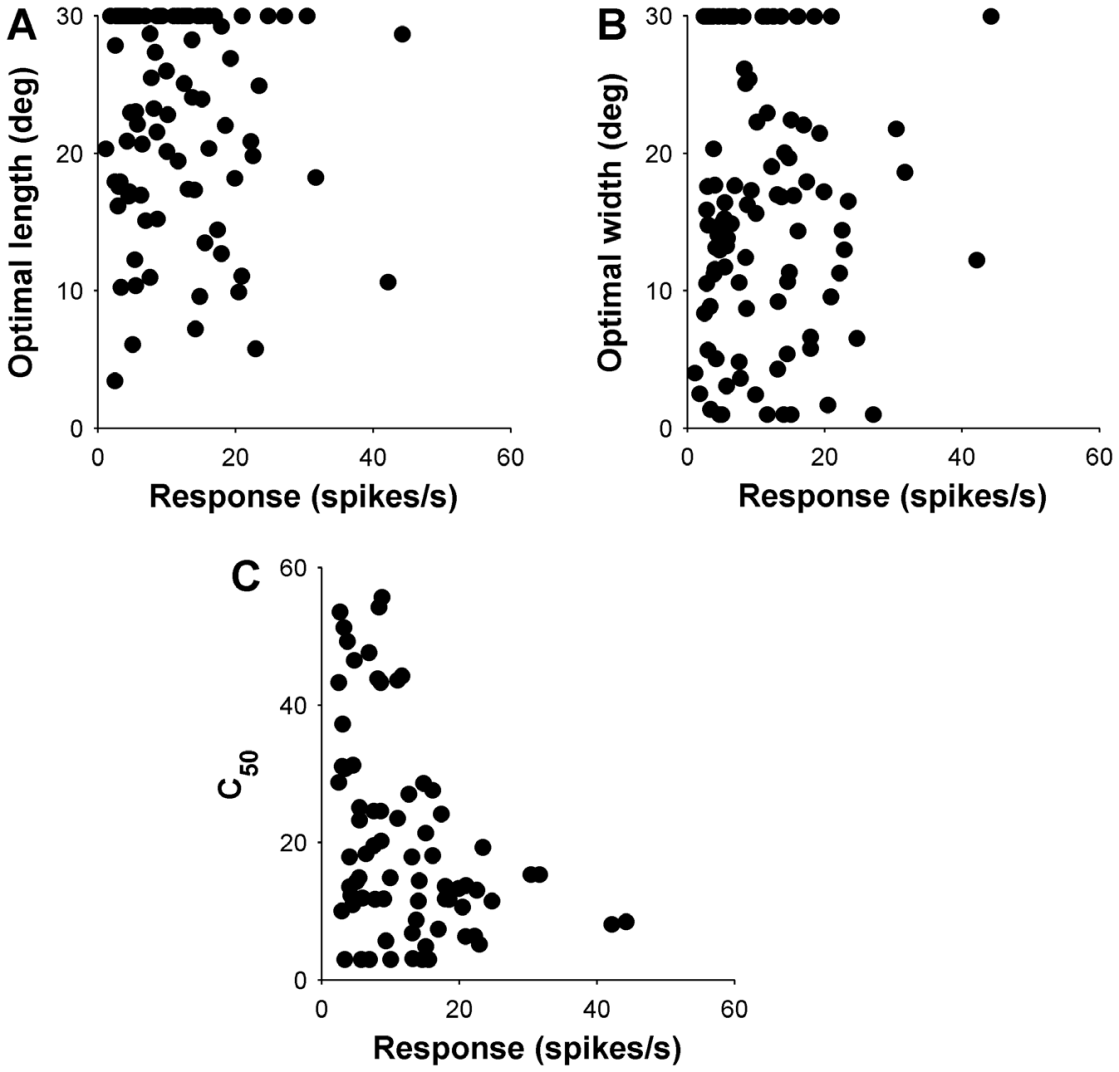
The effects of response strength on preferred size, latency and C_50_ for DM. Top row illustrates the relationship between response and (A) optimal length and (B) optimal width. (C) Illustrates the relationship between response and C_50_ for DM.

**Figure 10:**
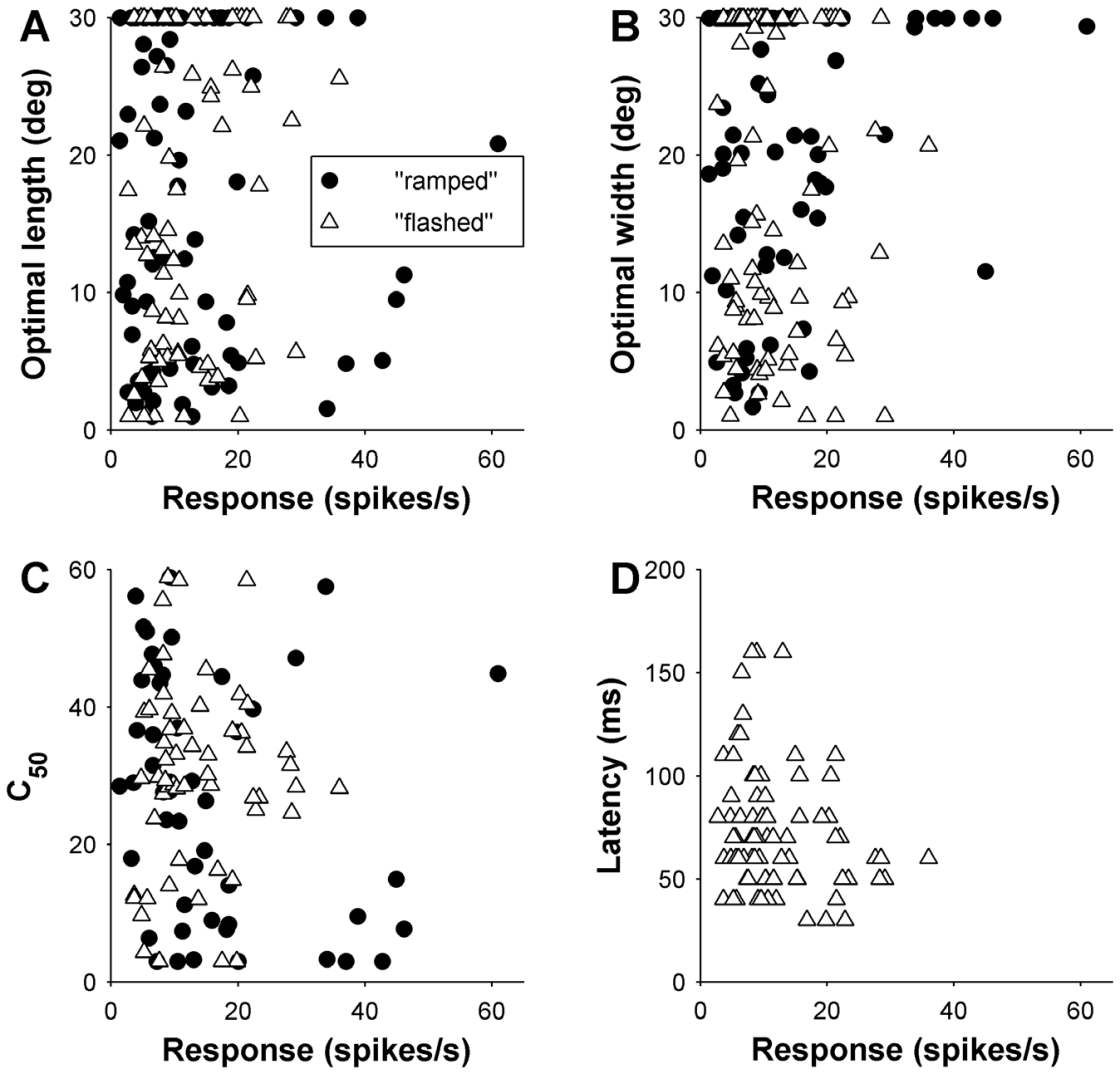
The effects of response strength on preferred size, latency and C_50_ for MT. Top row illustrates the relationship between response and (A) optimal length and (B) optimal width. The method of presentation is identified, see legend in (A). (C) Shows the relationship between response and C_50_while (D) illustrates the relationship between response and latency for MT.

We wanted to determine whether or not the relationships reported above could be explained by a covariation in firing rates. For DM, no significant correlation was found between maximum firing rate and preferred length (Fig. 9A; Spearman’s r = −0.30 P = 0.77) or width (Fig. 9B; r = 0.28 P = 0.78). However, a significant inverse correlation was found between firing rate and contrast sensitivity, as estimated by C_50_ (Fig. 9C; r = −0.37, P = 0.001). For MT, no significant relationship was found between maximum response rate and preferred grating length, either with the “ramped” (Fig. 10A; r = 0.11, P = 0.30) or the “flashed” (r = 0.21, P = 0.07) methods of presentation. In addition, no relationship was found between preferred width and maximal response rate (Fig. 10B; Ramped: r = −0.08 P = 0.50; Flashed: r = 0.00, P = 0.99). An inverse relationship was found between contrast sensitivity and response rates for MT, but only for the ramped method of presentation (Fig. 10C Ramped: r = −0.31 P = 0.03; Flashed: r = −0.03, P = 0.81). Lastly, no significant relationship was found between maximal response rate and latency (Fig. 10D; Flashed only: r = −0.23, P = 0.053). As the relationships between other parameters and response rate are at best sporadic, the relationships reported in the above sections are unlikely be attributed solely to co-variation with firing rates.

## Discussion

Based on single-unit responses recorded in two extrastriate areas (DM and MT), we report on the relationship between three basic response properties of visual cortical neurons: contrast sensitivity, size summation properties, and response latency. In both areas, neurons that were more sensitive to lower contrast preferred smaller (primarily, narrower) stimuli, and were more likely to show side inhibition when stimulated with gratings of up to 30°. Additionally we found that, in MT, cells with high contrast sensitivity tended to show shorter response latencies. Finally, shorter-latency cells in MT tended to summate over smaller expanses of visual space (again, primarily along the stimulus width dimension), in comparison with neurons that had longer latencies.

### Relationship between Contrast Sensitivity, Latency and Spatial Extent Properties

Unlike earlier studies, we did not measure size summation and inhibition properties at different contrasts for the same cell [1], [5]. Rather, we asked whether the size summation properties of different neurons in a population, assessed using stimuli of a same contrast, were related to their contrast sensitivity. In general, our results imply that a proportion of the variance observed in the size-summation and surround inhibition properties of DM and MT cells is related to the variance of contrast sensitivity across the population. These relationships were relatively modest, accounting for less than 15% of the total variance, suggesting other factors are also involved in shaping receptive fields. However, these co-variations provide insights into additional mechanism that can be incorporated into current models of cortical population responses, as discussed below.

We also found in MT cells that were sensitive to lower contrasts also responded with shorter latencies. This result is in agreement with many previous reports spaning several visual areas of the brain [8], [9], [10], [12], [45]; in fact, this effect becomes even stronger in higher-order association cortices [48]. Predictably, given the relationship between contrast sensitivity and size summation, a corresponding relationship between latency and size summation was also found: MT cells that preferred smaller gratings had shorter latencies.

A previous investigation of the relationship between a number of stimulus parameters and latency in MT found that cells which show obvious spatial inhibition have longer latencies [45], the opposite of what we observed. However, the same study also found this relationship can be attributed to a large extent to covariation with response strength. While some effects of response strength were evident, our data suggest that the relationships between contrast sensitivity, latency and size summation cannot be explained solely on the basis of a covariation (Figs. 9 and 10). The difference between our results in area MT and those of Raiguel et al. [45] can be directly attributed to the type of stimuli used: while we used near-optimal sine-wave gratings, the previous study employed fields of random dots, with distinct discontinuities in luminance which contained multiple spatial frequencies. Interestingly, they also found that the relationship between latency and response strength depended on the type of stimuli used, and others have also found differences in MT receptive field properties when using stimuli of single and multiple spatial frequencies [49]. Altogether, these observations suggest that a model based solely on “feedforward” connections cannot account for the results.

### Neural Mechanisms

Our initial hypotheses were based on a hierarchical model, whereby larger receptive fields would require convergence of information from larger pools of “feedforward” afferent neurons. Given that anatomical studies suggest that areas DM and MT occupy the third hierarchical processing level in the marmoset visual cortex [22], [26], the initial computations by neurons within these areas are presumably dependent on information sent by V1 and V2 cells, while further processing is likely to be based on intrinsic connections, or feedback from other extrastriate areas [50]. Some aspects of the present data seem to conform to these expectations. For example, in MT cells showing larger receptive fields had longer response latencies, and higher C_50_ values. Conceptually, these observations parallel differences observed between the granular and supragranular layers of V1, or between V1 and V2 [46], [51], [52], [53]. However, our data suggest that these relationships are based on mechanisms that are more specific than simple hierarchical convergence. First, they indicate that the interdependencies between latency and contrast sensitivity on receptive field size are more evident with respect to variations in the receptive field width, rather than receptive field length. Thus, there is an anisotropy in the cellular interactions that give rise to these effects. Second, they show that the type of size selectivity that underlies our data originates, in part, from inhibitory interactions: cells showing side-inhibition to stimulation of regions up to 15° on either side of the receptive field centre were more likely to respond at shorter latencies, and to have low C_50_ values. Both of these new observations need to be incorporated in models of the circuitry of visual cortex aimed at describing population responses to complex visual stimulation.

Previous work has demonstrated that probing the responses of a same neuron with stimuli of increasingly higher contrasts reveals progressively smaller summation areas, and greater surround inhibition. This led to the hypothesis that, within the neural population of a given area, cells that were more sensitive to contrast would tend to reach peak responses upon presentation of relatively small stimuli. A relationship between different spatial summation and contrast sensitivity properties was reflected in our data, albeit, perhaps surprisingly, only with respect to the width dimension of the gratings. Our results in this respect are, in principle, compatible with those of Pack et al. [5], who have investigated the analogous relationship in macaque area MT, using circularly symmetrical stimuli. In contrast, a study in area V1 has described that an increase in contrast specifically increased the incidence of end-inhibition, resulting in reduced receptive field sizes along the length dimension [3]. A relationship between optimal length and contrast sensitivity was not reflected in our population data, on extrastriate areas.

Contrast-dependent changes in the spatial structure of receptive fields have been previously reported for neurons in various early stages of visual processing [1], [2], [3], [4], [6], [12], making it possible that the effects we observed in MT and DM in part reflect computations performed in earlier areas. However, the spatial extent of the inhibitory effects observed among many cells in both MT and DM far exceeds that observed in V1 [54]. It has also been suggested that contrast-dependent modulation of responses in V1 depends in part on feedback from MT [55], making it more likely the current observations are at least partly due to neural mechanics within DM and MT, and perhaps beyond (see [5] for discussion).

Historically, gain normalization (a mechanism whereby the activity of neurons is normalized, or divided, by the activity of a general pool of neurons representing neighbouring receptive fields) has been successful in explaining the non-linear relationship between neuronal firing rates and stimulus contrast [56]. More recently, the same mechanism has been used to explain complex, non-linear and non-retinotopic spatial summation effects in MT neurons [5], [17]. It is possible that spatial summation and contrast sensitivity share the same gain normalization mechanisms in the cortex. As neurons in the normalization pool will respond differently to sine-wave gratings and random dot patters, gain normalization can also explain differences in spatial summation properties between stimuli (our results and those of [45]): the activity of the normalization pool will cause the spatial summation properties to change, thus affecting the activity of the recorded cell, even though feedforward connections remain the same.

Our observation that the relationship between contrast sensitivity and size summation is dependent on receptive field dimension has implications for the likely composition of normalization pools. Specifically, the difference between length and width summation properties suggests that the “shape” of the normalization pools (i.e., the composition of the neuronal population which, via intrinsic connections, participates in this process) may be more specific than first thought. For example, the rate of which neurons in the pool are recruited is likely to be dependent on the direction of interactions across the topographic maps of DM and MT. The concept of a more specific normalization pool is not new, having been used to explain the network behaviour of neurons in response to the presentation of plaid stimulus. In that case, the range of MT responses were attributed to the contribution of a “tuned” normalization pool consisting of cells that respond well to a particular direction of motion, versus a more generalized “un-tuned”, non-specific normalization pool, which encompasses all cells regardless of their direction preference [57]. A similar concept, applied with respect to visual (hence cortical) space, could account for our current observations. Given that cortical areas in which neurons have very different size summation properties (DM and MT) display the same relationship between size selectivity and contrast sensitivity, this may reflects a more general mechanism for motion-sensitive areas (see below).

### Comparison between DM and MT

Our data extend earlier reports of functional differences between cells in areas DM and MT, two subdivisions of extrastriate cortex that receive strong inputs that originate from Brodmann’s “layer 4b” of V1 (layer 3c in Hassler’s nomenclature; see [58]). Whereas the V1 input to MT originates almost exclusively from this layer, cells in DM also receive projections from cells located in other subdivisions of supragranular V1 [59]. In addition, while the extrastriate projections to MT reveal a heavy bias in favour of dorsal stream motion-processing areas, the projections to DM reveal a wider variety of influences, including significant projections from ventral stream areas [26]. Our results demonstrate some important functional differences between these areas, but also highlight similarities.

Through systematic tests using identical procedures, we confirmed the suggestion [17] that neurons in these areas are distinct in terms of selectivity to stimulus size: DM cells tend to summate more extensively along the receptive field length dimension, and tend to show a stronger degree of side-inhibition, while MT cells tend to summate along the receptive field width, and show on average stronger end-inhibition (at least, for stimuli extending up to 15° on either side of the receptive field centre). These findings add to the previously described functional distinctions between these areas, in terms of the relative prevalence of direction and orientation selectivity [25], [28]. Nonetheless, the distributions of mid-saturation contrast (C_50_) revealed no significant difference between the DM and MT samples. Moreover, in both areas, cells that were highly sensitive to contrast reach maximal responses upon presentation of relatively narrow gratings, whereas a corresponding relationship was not obvious with respect to stimulus length (Figs. 5 and 6).

The distinct size preference of neurons is likely to be related to functionality. Area MT has been described as an integrator and segregator of motion (see [60]). Thus, it is fitting that the optimal stimulus integration window is larger along the axis of motion (grating width), and that this dimension reveals less spatial inhibition, since motion in most naturalistic situations is smooth and continuous [61]. The minority of cells that are inhibited along the axis of motion may serve to code for the beginning or termination of motion, acceleration, or changes of direction or speed. The observed increased contrast sensitivity and shorter latencies for cells that have restricted receptive fields along this dimension may aid in highlighting such discontinuities in motion. Conversely, the more commonly observed cells, which lack obvious inhibition along the axis of motion, will be able to capture more motion energy, and may not require as high a level of sensitivity to low contrasts.

Cells in area DM, on the other hand, tend to prefer long contours, with relatively little inhibition along the length of the grating, and the frequent occurrence of facilitation beyond the receptive field. Along with its narrow orientation tuning [28], its receptive fields are well suited to indicating the continuity of borders of larger objects with accuracy. The increased contrast sensitivity of narrow receptive fields, including those with side-inhibition, may serve to enhance the contours of objects. Conversely, the minority of cells that lack side-inhibition may be optimized for integration rather than detection. It is worth noting that, in this area, cells that prefer wider grating also have coarser orientation tuning [28], again pointing to a role in detection and integration across space, rather than fine analysis of borders.
